# Range Expansion and Population Dynamics of an Invasive Species: The Eurasian Collared-Dove (*Streptopelia decaocto)*

**DOI:** 10.1371/journal.pone.0111510

**Published:** 2014-10-29

**Authors:** Spencer N. Scheidt, Allen H. Hurlbert

**Affiliations:** Department of Biology, University of North Carolina, Chapel Hill, North Carolina, United States of America; Hungarian Academy of Sciences, Hungary

## Abstract

Invasive species offer ecologists the opportunity to study the factors governing species distributions and population growth. The Eurasian Collared-Dove (*Streptopelia decaocto*) serves as a model organism for invasive spread because of the wealth of abundance records and the recent development of the invasion. We tested whether a set of environmental variables were related to the carrying capacities and growth rates of individual populations by modeling the growth trajectories of individual populations of the Collared-Dove using Breeding Bird Survey (BBS) and Christmas Bird Count (CBC) data. Depending on the fit of our growth models, carrying capacity and growth rate parameters were extracted and modeled using historical, geographical, land cover and climatic predictors. Model averaging and individual variable importance weights were used to assess the strength of these predictors. The specific variables with the greatest support in our models differed between data sets, which may be the result of temporal and spatial differences between the BBS and CBC. However, our results indicate that both carrying capacity and population growth rates are related to developed land cover and temperature, while growth rates may also be influenced by dispersal patterns along the invasion front. Model averaged multivariate models explained 35–48% and 41–46% of the variation in carrying capacities and population growth rates, respectively. Our results suggest that widespread species invasions can be evaluated within a predictable population ecology framework. Land cover and climate both have important effects on population growth rates and carrying capacities of Collared-Dove populations. Efforts to model aspects of population growth of this invasive species were more successful than attempts to model static abundance patterns, pointing to a potentially fruitful avenue for the development of improved invasive distribution models.

## Introduction

Invasive species, though considered by many to represent a significant threat to global biodiversity [1], [2], can provide a unique opportunity to study ecological and evolutionary processes on a scale that is otherwise infeasible and potentially unethical [3]–[5]. In particular, studying a species as it spreads across a broad geographic region can provide a unique context for examining the primary factors influencing distribution, abundance, and population dynamics. In cases where the invasion and spread are ongoing, such analyses can be used to make predictions about future distributions and the environmental features that may be facilitating population spread and establishment [6]. However, despite several classical examples [7]–[9], ecologists still lack a comprehensive predictive model of invasion dynamics [4], [10].

Even though species invasions are relatively common and widespread [11], [12], few invaders are ideal for ecological study. First, most invasions either occur without human knowledge or are not noticed until well after initial colonization and establishment. Second, because many invasive species are only detectable by scientists or natural resource managers, the number of records may be few and patchily distributed, especially during the early stages of invasion. Avian invaders, however, are much less likely to escape the notice of the legions of amateur birdwatchers constantly on the lookout for rarities, and semi-standardized surveys such as the North American Breeding Bird Survey and the Christmas Bird Count provide quantitative indices of abundance in addition to the more commonly available information on presence-absence over a broad geographical extent. It is thanks to the efforts of these types of surveys that the invasion of North America by one species, the Eurasian Collared-Dove (*Streptopelia decaocto*), has been documented in detail over the past forty years [13], [14].

Originally a native of India, the Eurasian Collared-Dove (hereafter the ECD) was introduced into the Bahamas in the mid-1970s and has spread rapidly throughout the continental United States in the past four decades in a similar manner to its invasion of Europe earlier in the 20th century [13], [15]–[17]. A known grain forager, the ECD has been linked to human settlements and non-intensive agricultural zones in several large scale ecological studies in both the U.S. and Europe [16], [18]–[20]. Researchers have also found negative relationships with forest cover and areas of intensive agriculture [16], [21], [22]. With respect to rates of spread, Hooten and Wikle (2008) used a hierarchical Bayesian diffusion model and found higher rates of diffusion in the western U.S. compared to regions closer to the point of origin of the invasion. Higher rates of spread could be due either to landscape features that facilitate dispersal, or to local environmental conditions that allow greater population growth and the production of more propagules. However, because the ECD exhibits a characteristic jump dispersal pattern, often dispersing great distances and ‘backfilling’ areas in-between [21], and inhabits a wide climatic range, identifying clear patterns in its invasive spread has proven difficult [19], [20], [23], [24]. In addition, local sites vary in the length of time that ECDs have been present, from sites that have only recently been colonized to areas that have been continuously occupied for decades. The fact that the abundance surface of the ECD is inherently non-equilibrial may be part of the reason why studies that have attempted to explain spatial variation in abundance from a snapshot in time have done so with limited predictive power [20].

Here, we make use of historical information on abundance from several hundred locations throughout North America to evaluate how local population trajectories of an invasive species vary across its expanding range. By fitting simple population growth models (exponential, quadratic and logistic), we are able to estimate parameters such as population growth rate and carrying capacity (for sites that are no longer increasing) for many local sites. We are then able to assess the importance of climate, land cover, and historical or geographic variables for explaining these two parameters, and make the following predictions. First, sites close to the point of invasion (i.e. south Florida) with older populations are most likely to have reached carrying capacity, while sites along the range expansion front are more likely to still be in a phase of exponential growth. Second, while distance from the point of invasion and population age should be predictors of ECD abundance in any one year [19], [20], these historical variables should be less important in explaining local carrying capacities at sites that show signs of having reached an equilibrium. Third, based on previous literature [18], [21], [22], it is predicted that areas associated with developed and agricultural land cover will be positively correlated with growth rate and carrying capacity, while forested land cover is predicted to be negatively correlated with these parameters.

## Methods

Abundance data for the ECD up through 2010 were obtained from two publicly available datasets: the North American Breeding Bird Survey (BBS) [25], and the Christmas Bird Count (CBC) [26]. Each BBS route consists of 50 3-minute point counts spaced along a 39.4 km roadside route that are conducted on a single day during the breeding season (typically in June), over which a single observer records all birds seen or heard within 400 m of each point. Data that did not meet BBS quality standards (due to inclement weather, inappropriate survey times, etc.) were removed from our analysis. Abundance counts from all 50 stops at each individual BBS route were combined into a single metric of abundance for that particular route. Each CBC survey consists of a circular region 24 km in diameter in which variable numbers of observers count all of the birds seen or heard over the course of the entire day in late December or early January. To account for variation in survey effort through time and across count circles, we divided these raw counts by the number of party hours (i.e. the total number of hours independent birding parties spent counting birds on that day). These two surveys are conducted at distinct times of year and at very different grain sizes, and as a result may provide different insight into the ECD invasion.

Traditionally, studies have attempted to explain static abundance patterns across the range, and so we analyzed the abundance surface of the ECD for a single year, 2010, across 792 CBC sites that reported the presence of the ECD. Our main focus, however, was on characterizing how abundance has varied through time at local sites across the range, an approach we hypothesized would have more explanatory power than the static approach. Logistic, exponential, and quadratic models were fit to population trajectories for each route (BBS) or count circle (CBC) with at least 8 years of abundance data; 107 BBS routes and 292 CBC count circles fit these criteria. Quadratic functions were fit because a number of empirical patterns were hump-shaped, and the quadratic allows an estimate of an upper limit to carrying capacity. Logistic curves were fit using maximum likelihood, although models failed to reach convergence for 10 BBS and 31 CBC sites for which a logistic was a poor empirical fit. Because the ECD was known to be absent from all sites initially, sites that were best fit by monotonically decreasing functions (negative exponential, negative linear, or negative logistic; a total of 14 BBS and 36 CBC sites) were presumed to have missed the dynamics of initial colonization and population growth and were discarded from further analyses. These negative growth rate model fits could be due to birders not recording the presence of the ECD during the early years of the invasion. The similarity of the ECD to the Ringed Turtle-Dove (*Streptopelia risoria*) combined with its rapid spread has been a point of confusion for many birders, at least until they became aware of the ECD’s presence and appearance [23].

Growth model fit was assessed using the small sample-corrected Akaike Information Criterion (AIC_c_) [27], for each model in each location. The model with the lowest AIC_c_ score was presumed to be the best fit, although sites that had ΔAIC_c_ scores of less than 2 indicate that other models also had some measure of support [27]. Exponential growth rates were estimated using linear regression on log-transformed abundance values for all sites where ΔAIC_c_ for the exponential model was less than 2. Local carrying capacity was estimated in one of two ways. For sites that were best fit by quadratic models, the maximum abundance value where the derivative equaled 0 was identified as an upper limit on carrying capacity. For population trajectories best fit by a logistic model, carrying capacity was taken as the asymptote parameter. Analyzing these two types of carrying capacity separately had little effect on model results, and so only the combined analysis is presented and the term “carrying capacity” is used to refer to both. Sites that were best fit by an exponential model but that had a quadratic or logistic ΔAIC_c_ score of less than 2 were included in the carrying capacity analysis using the value of carrying capacity from whichever of the two models had the lowest ΔAIC_c_ score. After imposing these filters, 50 BBS routes and 136 CBC circles were used in the analysis of carrying capacity, and 58 BBS routes and 187 CBC circles were used in the analysis of population growth rate.

We examined three categories of predictors of carrying capacity or growth rate: historical/geographical, land cover, and climate variables. Historical/geographical variables include invasion distance and population age. Invasion distance was estimated as the great circle distance between each local site and the initial point of introduction for each respective data set (Florida route 36 in 1986 for the BBS and Bahamas count circles BASA and BASC in 1985 for the CBC) [6], [20]. It should be noted that this metric ignores natural boundaries to dispersal (such as the Gulf of Mexico separating south Florida from south Texas), but is expected to be a reasonable approximation for total land-based distance. Population age was estimated as the number of years since ECDs were first observed at a local site. This metric assumes that the ECD was immediately detected after its initial colonization.

Land cover data were extracted from the National Land Cover Dataset (NLCD) 2006 raster, which covers the conterminous U.S. with 16 land cover classes at a resolution of 30 m, where each pixel is assigned to only one land cover class [28]. BBS routes were clipped with a 400 m buffer along their entire length (39.4 km), which corresponds to the survey area of the route. CBC sites were clipped with a 24 km diameter buffer. Land cover percentage values were calculated by dividing the number of pixels in each land cover class by the total number of pixels in the buffer and multiplying by 100. Because the ECD is known to associate with human settlement [16], [18]–[20], variables reflecting different types of developed and agricultural land cover were selected for further analysis. In addition, researchers have documented negative relationships between ECD prevalence and forest cover [16], [21], [22], and so an aggregated measure of forested cover was also included in the analysis. Specifically, we calculated the percentage of land cover surrounding focal BBS routes and CBC circles in each of the following NLCD categories: open (<20% impervious cover), low intensity developed (20–49% impervious cover), medium intensity developed (50–79% impervious cover), high intensity developed (>80% impervious cover), pasture (pasture or hay accounts for ≥20% of vegetation), cropland (crops account for ≥20% of vegetation), and forest (≥20% tree cover).

Climate data were extracted from the WorldClim BioClim data set (20 arc minute resolution) [29], over the same spatial buffers around BBS and CBC sites. Preliminary univariate analyses allowed us to limit the number of climate variables to two strong predictors that would affect primary productivity, average habitat suitability and environmental tolerance: mean annual precipitation (mm) and mean annual temperature (°C), each averaged over the appropriate buffer. The distributions of land cover and climate variables encompassed by the BBS and CBC datasets are shown in Figure S1, and differences between the distributions were assessed with Mann-Whitney two-sample rank sum tests.

Our goal was to develop predictive models of ECD population carrying capacities and growth rates using these three classes of predictor variables, and to assess the relative importance of those predictors. As a preliminary analysis, we utilized regression trees to identify potentially important interactions between predictor variables [30]. No strong interactions were found, with the exception of CBC invasion distance and CBC low intensity percent developed cover in our growth rate analysis, which we ignored because it increased the AIC score of our CBC growth model. Because all of the predictor variables in our three categories had *a priori* support, we considered the set of linear models representing all possible combinations of those eleven variables as main effects for five distinct analyses: 1) predicting carrying capacity using BBS data, 2) predicting carrying capacity using CBC data, 3) predicting 2010 abundance using CBC data from 2010, 4) predicting population growth rate using BBS data, and 5) predicting growth rate using CBC data. All variables and responses for each dataset were independently mean centered at 0 and scaled so that the standard deviation was equal to 1. This normalization facilitates the comparison of parameter estimates across predictors that vary in units and scale. Because the distributions of environmental variables differed slightly between BBS and CBC datasets (Figure S1), we also conducted the above analyses on jointly standardized variables in which values from the BBS and CBC sites were combined prior to normalization. Models were evaluated in an information theoretic context, and for each linear model in a set, the AIC_c_ score, R^2^ and Akaike weight were computed. For predictive purposes, we used model averaging in which parameter estimates for each variable are averaged across all models in which that variable occurs, weighted by each model's Akaike weight. Model averaging provides a more precise and less biased inference about the predicted effects of a set of variables, especially when no one model has overwhelming support [27]. Finally, we calculated individual variable relative importance weights (*w_i+_*), which are a measure of how important a variable is relative to the other variables considered, by summing the Akaike weights of all models containing that particular variable (Burnham and Anderson 2002). A variable relative importance weight close to 1 indicates that that variable tends to be included in the models that collectively have the highest levels of support.

For each data set and response variable (population growth rate or carrying capacity), a linear model containing all of the weighted parameters (see Table 1) was evaluated for potential over-fitting using Leave-One-Out cross validation. Cross validation is a common model evaluation technique that divides a data set into training and validation segments [31], and can be used to assess how well the training data predict the validation data [32]–[34]. Leave-One-Out-cross-validation was applied to each model, where *n*-1 data points are used to predict the remaining datum *n* times to create an unbiased estimate of average prediction error. This prediction error was used to calculate R-squared inflation due to overfitting of the data set for each model. Finally, because cross-validation may overestimate the goodness of fit of models when model residuals are spatially autocorrelated [35], we plotted Moran’s I correlograms using the ncf package in R [35] for our four main dependent variables (growth rate and carrying capacity for each of the two datasets), and for their respective average model residuals.

**Table 1:** Variable relative importance weights and weighted parameter estimates.

	*K*BBS		*K*CBC		*NCBC: 2010*		*r*BBC		*r*CBC	
	*wi+*	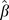	*wi+*	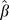	*wi+*	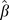	*wi+*	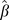	*wi+*	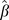
*Historical/geographical variables*	0.29	−0.02	0.28	−0.03	0.35	0.02	**0.98**	**0.59**	**1.00**	**0.59**
Distance to invasion origin	0.23	−0.01	**0.99**	**0.40**	**1.00**	**0.29**	0.31	−0.04	0.26	0.00
Time since colonization										
*Land cover variables*	0.29	−0.03	0.63	−0.12	**0.95**	(0.14	0.24	0.01	0.29	(0.01
Developed: open cover	0.55	(0.19	0.61	0.16	0.91	**0.15**	0.30	0.01	**0.76**	**0.15**
Developed: low intensity	**0.99**	**1.27**	0.33	0.01	**0.82**	**−0.11**	0.48	0.18	0.37	0.03
Developed: med. Intensity	**0.93**	**−0.84**	0.48	0.08	0.37	0.01	0.36	−0.05	0.38	0.04
Developed: high intensity	0.42	−0.08	0.37	−0.03	0.49	−0.03	0.25	0.01	0.59	−0.06
Forest	0.39	−0.06	0.49	−0.05	0.35	−0.01	**0.93**	**−0.29**	0.26	0.00
Pasture	0.22	0.00	**0.99**	**0.27**	0.43	0.02	0.34	−0.04	0.32	0.01
Crops										
*Climate variables*	0.35	0.05	**1.00**	**−0.54**	**1.00**	**−0.34**	0.25	0.02	0.35	−0.04
Avg. Precipitation	0.51	0.13	**0.76**	(0.18	0.65	(0.05	0.60	0.16	0.57	(0.07
Avg. Temperature										
Model Averaged R2	0.48		0.35		0.17		0.41		0.46	

All derived data used both in model selection and cross validation are archived in the Dryad Digital Depository.

## Results

**Figure 1:**
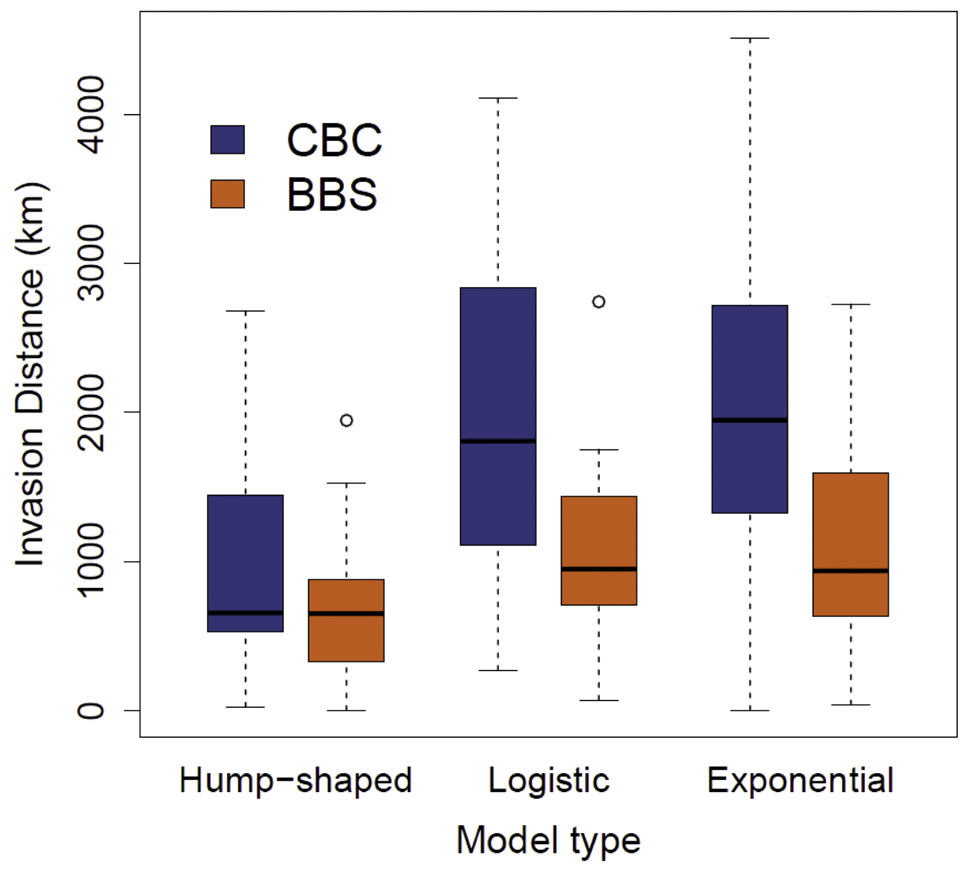
Invasion distance and model type. Boxplots of distance of a site from the original introduction point grouped by population trajectory type for the Christmas Bird Count (blue) and the Breeding Bird Survey (orange).

**Figure 2:**
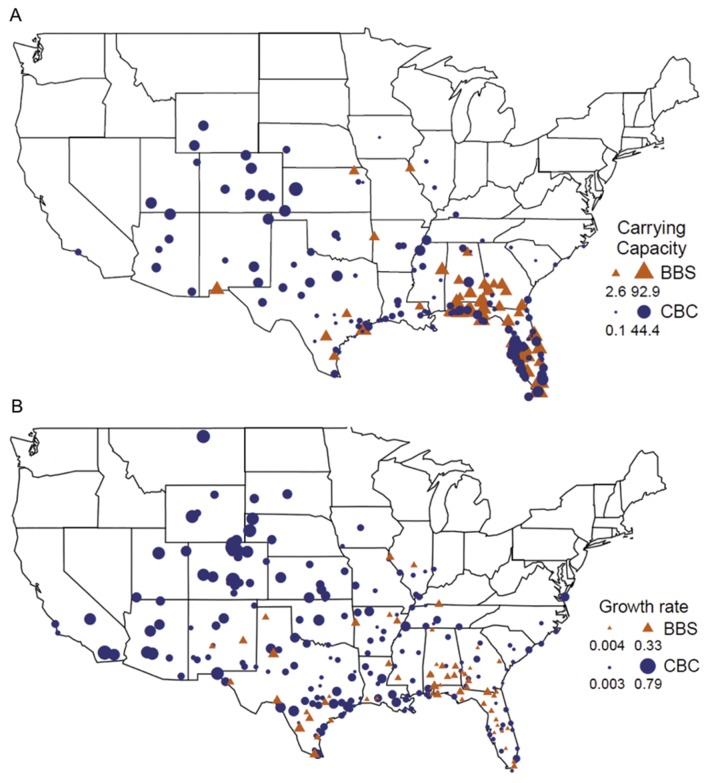
Carrying capacities and growth rates. Map of all (a) Christmas Bird Count circles (blue) and Breeding Bird Survey routes (orange) used in carrying capacity analysis scaled by estimated carrying capacity, *k* (b) Christmas Bird Count circles (blue) and Breeding Bird Survey routes (orange) used in growth rate analyses with the sizes of each location’s point scaled by estimated population growth rate, *r*. Minimum and maximum symbol sizes and their meanings are given in the legend of each figure panel.

In both BBS and CBC data sets, the type of population model (exponential, quadratic, or logistic) receiving the greatest support at a particular site varied as a function of the distance to the original invasion point (BBS: p = 0.003; CBC: p<<0.001) and of the time since colonization (BBS: p = 0.007; CBC: p<<0.001; Figure 1, Figure 2). Hump-shaped population trajectories occurred at sites with the oldest populations and that were located closest to the point of invasion, while most of the younger populations out on the expansion front exhibited exponential growth. Sites best fit by logistic growth are intermediate on average in both age and distance.

### Carrying capacity

**Figure 3:**
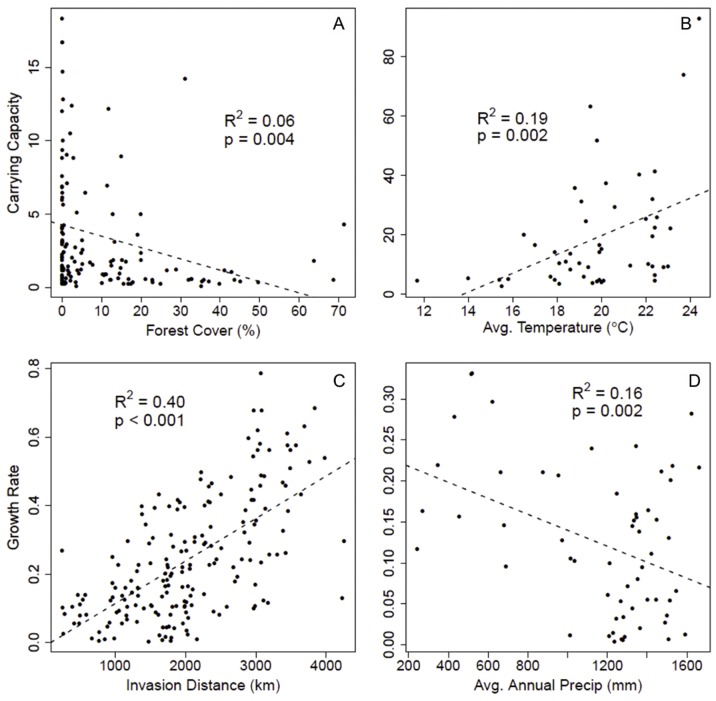
Linear predictors of carrying capacities and growth rates. Univariate plots of the strongest predictor variables for: (a) Christmas Bird Count (CBC) carrying capacity versus percent developed low intensity cover, (b) Breeding Bird Survey (BBS) carrying capacity versus minimum temperature of the coldest month (°C); and population growth rate: (c) CBC population growth rate versus invasion distance, (d) BBS population growth rate versus average annual temperature (°C).

The variables most important for explaining spatial variation in carrying capacity differed for the BBS and CBC data sets (Table 1, Figure 3). Although the BBS model with the lowest AIC_c_ score described a moderate amount of variance (R^2^ = 0.46), its Akaike weight was only 0.03, and several other models had roughly equivalent support. As such, we evaluated overall variable importance weights rather than simply identifying which variables were included in the “best model”, and we interpret model-averaged parameters, which collectively explained 48% of the variance in carrying capacity. An examination of variable weights across all models indicated that medium intensity developed cover (a positive relationship, *w_i+_* = 0.99) and high intensity developed cover (a negative relationship, *w_i+_* = 0.93) were the variables with the strongest effects on carrying capacity in the BBS data set (Table 1).

The CBC model using model averaged parameter estimates explained 35% of the variance in carrying capacity. Based on variable relative importance weights, the strongest positive predictors were time since colonization (*w_i+_* = 0.99) and cropland cover (*w_i+_* = 0.99), while the strongest negative predictor was precipitation (*w_i+_* = 1.00), with a moderate negative effect of average temperature (*w_i+_* = 0.76, Table 1). For comparison, we also modeled spatial variation in observed abundance from a single year, as opposed to carrying capacity estimated from the available time series, for 792 count circles in 2010. As in the carrying capacity analysis, time since colonization and average precipitation were two of the most consistent predictors of single-year abundance, with differences in which land cover variables were identified as most important (Table 1). Most importantly, however, the variance in single-year abundance was inherently noisier (R^2^ = 0.17), with the model averaged parameters accounting for less than half of the variation explained in carrying capacity.

### Population Growth Rate

Similar to the carrying capacity results, the collective model averaged BBS and CBC models incorporated different predictors (Table 1). The model averaged BBS growth rate model explained 41% of the variance in growth rate. Variable relative importance weights point to a strong positive relationship with distance to the point of invasion origin (*w_i+_* = 0.98), and a negative relationship with the amount of pastureland (*w_i+_* = 0.93, Table 1). Total explained variation in population growth rate based on CBC data was similar to the BBS analysis (model averaged R^2^ = 0.46), and distance to invasion origin was again the strongest predictor (*w_i+_* = 1.00). However, for the CBC, low intensity developed cover was the most supported land cover variable (*w_i+_* = 0.76, positive relationship) while pastureland appeared to be unimportant (Table 1).

### Cross Validation, Spatial Autocorrelation and Variable Standardization

As expected, Leave-One-Out-cross-validation detected model over-fitting, although the extent of the average prediction error and the degree of reduction in R-squared varied from model to model (Table 2). In general, cross validation suggested that our models still accounted for a large percentage of the variation in both carrying capacity and growth rate. Both the BBS and CBC growth rate models experience R-squared decreases of less than 10%, and still explained between 37 and 45% of the variance (Table 2). Cross validation of carrying capacity models exhibited larger decreases in R-squared (between 29 and 36%), although both still explained 150–200% of the variation in carrying capacity compared to the cross-validated single year abundance model (Table 2).

**Table 2:** Cross Validation Results.

	Original Results		Leave-One-Out cross validation		% R^2^ decrease (LOOCV)
	MSE	R^2^	MSE	R^2^	
*Model*					
*K*_BBS_	0.67	0.48	0.83	0.34	29.1
*K*_CBC_	0.70	0.35	0.84	0.23	36.1
*N_CBC: 2010_*	0.84	0.17	0.85	0.16	6.0
*r*_BBS_	0.73	0.41	0.76	0.37	9.5
*r*_CBC_	0.57	0.46	0.58	0.45	3.0

Leave-One Out-cross-validation (LOOCV) results for the model averaged main effects models predicting carrying capacities and growth rates of the BBS and CBC data sets. The mean square error (MSE) and R^2^ values are provided for the original, uncorrected models and the corrected LOOCV models, with their associated R-squared shrinkage values.

Raw values of carrying capacity and population growth rate exhibited varying degrees of spatial autocorrelation (especially growth rates). However, this was accounted for primarily by spatial autocorrelation in the predictors, and the residuals of our averaged models exhibited very little autocorrelation even at the closest lag distances (Figure S2). As such, spatial autocorrelation should not influence our interpretation of goodness-of-fit from the cross-validation results.

In our carrying capacity and population growth rate analyses, independent standardization of the BBS and CBC datasets had minimal impact on our model-averaged results compared to the analysis in which both datasets were combined prior to standardization. No parameter estimates changed sign and all variable relative importance weights were identical to within 0.01 (see Table 1 and Table S1).

## Discussion

Examining the invasive spread of the ECD by modeling population trajectories at individual sites across the range reveals novel aspects of the range expansion of this species. With both data sets we found that the best fitting growth models varied in a similar fashion with invasion distance and population age: hump-shaped distributions tended to fit older populations close to the initial introduction point, logistic distributions fit middle-aged populations at moderate invasion distances, and exponential models fit younger populations closer to the invasion front. This repeated pattern suggests that populations of the ECD may frequently go through three phases during colonization and establishment: (1) exponential increase, (2) a leveling off at or above local carrying capacity, and (3) a subsequent decline. Although the reasons for this decline are beyond the scope of this study, this may be evidence that populations of the ECD routinely overshoot the carrying capacity of local habitats. Another possibility is that these population trajectories could be depicting a traveling invasion wave that is initially fed by dispersing birds along the invasion front; once these dispersers pass through, declines in local abundance result. Regardless of why such a decline may occur, it seems reasonable to expect that populations of the ECD are more likely to show evidence of a carrying capacity as they age.

While the overall shape of population trajectories was related to historical and geographical variables, local carrying capacities and population growth rates varied in a predictable manner with land cover and climate variables across the ECD’s geographic range. As confirmed by cross validation analyses, our main effects models were able to explain at least 23–45% of the variance in our two main response variables (Table 2). The predictive ability of models for these population parameters was much greater than for the model attempting to predict static single year abundance values across the range. The fact that the strongest predictor of abundance in 2010 was population age confirms our prediction that abundance in any given year is more influenced by invasion history than is a site's estimated carrying capacity. Given the non-equilibrial nature of this system, the estimation of population parameters from modeling growth trajectories therefore facilitates the development of more predictive models that can link the occurrence of ECD to characteristics of the local environment.

Our results show that both land cover and climate are important factors for explaining the observed patterns. Developed land cover in different forms (BBS: medium and high intensity developed; CBC: open and low intensity developed) was a consistently strong predictor across both data sets for predicting variation in carrying capacity and growth rate. Given the previously known propensity of the ECD to exploit bird-feeders and grain as sources of food and use telephone poles, suburban trees, and buildings for roosting [17], its seems likely that the ECD is numerically responding to these basic habitat and energetic constraints. Many of our analyses found negative relationships with both open and high intensity developed cover, indicating that neither golf courses and large lawns, nor heavily urban centers offer the ECD the resources necessary to achieve high abundances or growth rates. In contrast, both low and medium intensity developed cover (20–80% impervious surface) typified by single family housing areas enhanced carrying capacity and population growth rates, depending on the data set. These results parallel observations that link the ECD to suburban land cover but find it is not present in dense urban areas [21], [23]. We also found that both forested cover and pastureland had weak negative effects on carrying capacity and population growth rate, consistent with previous studies [13], [21], [22], while cropland may enhance ECD populations in some cases (Table 1). This suggests that agricultural land cannot be aggregated when studying the ECD – pastureland does not offer the ECD much in the way of food or resources [17] – and may explain why other studies have not found stronger coarse scale relationships between agricultural cover and the ECD [19], [20].

Climate variables were also some of the strongest components of the best main effects models. In general, the ECD responded differently to climate depending on the data set being examined. Both of our BBS analyses found positive relationships with both precipitation and temperature, while all of our CBC analyses found negative relationships with the same variables. This could be due to differences in grain, spatial extent and sampling time, as discussed below.

Both carrying capacity and population growth rates were strongly related to land cover and climatic variables overall, but population growth rates were also affected by invasion distance to an equal extent in both data sets (Table 1). Population growth rates peaked at the expansion front (Figure 2), a result that parallels findings by Hooten and Wikle (2008) showing that the rate of spatial diffusion was greatest in the western U.S. We believe that populations at the invasion front could be experiencing higher growth rates for several reasons. First, rates of dispersal may be higher along the invasion front due to a lack of barriers to dispersal, especially dense forests. Second, sites closer to the original invasion point may be more likely to have begun approaching a population asymptote. Two sites might be on identical logistic trajectories, but the fitted estimate of population growth rate will be lower for sites that are farther along that trajectory. However, the correlation coefficient between growth rates calculated over a fixed time window (the first 8 years of data) versus growth rates calculated from the full time-series was quite high (*r* = 0.84), indicating that variation in time series length is not the primary source of variation in growth rates. Third, sites along the invasion front could offer more of the resources and habitats that facilitate the maintenance and growth of ECD populations. Regardless, populations of the ECD are increasing at a rapid rate far from the original point of introduction, which implies that the ECD is still on the path toward rapid range expansion.

Although the BBS and CBC datasets were similar with respect to the relative importance of broad classes of variables, the specific variables with the greatest support in our models differed depending on the data set. Several differences between the BBS and the CBC seem pertinent for explaining some of this variation. One obvious difference is that CBC surveys are conducted in winter, while the BBS is conducted in May or June. The ECD may exhibit seasonal behaviors that make individuals more or less likely to be detected in different seasons [36], and this might explain some of the discrepancies over the directionality of climate relationships between the two data sets. However, the ECD is capable of breeding year round throughout much of its North American range, and at this point no data suggest seasonal flocking or aggregation in the United States [17]. More importantly, the grain size of the two surveys differs by more than an order of magnitude: a CBC circle encompasses a total area of over 450 km^2^ while a BBS route covers only 25 km^2^. The larger sampling area of CBC surveys means that CBCs will be more likely to record ECD presence compared to BBS routes surveyed in the same location, especially when ECD densities are low. This explains the greater geographic coverage of the CBC relative to the BBS (and hence the greater overall distances from the invasion origin, Figure 1). This does not imply that the ECD is absent throughout the western U.S. during the breeding season, but rather that the number of sites on which it has been observed for a minimum of 8 years on BBS routes lags behind the number of CBC circles. This difference in spatial extent means that the CBC analysis covers a broader range of environmental conditions and reflects a different distribution of land cover values (Figures S1). For example, CBC sites tended to have less developed open land cover, pasture, and forest compared to BBS sites (Figure S1) which could potentially obscure or enhance the strength and direction of population parameter-environment relationships. The difference in CBC and BBS survey areas also means that environmental relationships are being evaluated at two distinct grain sizes, and it has long been appreciated that species-habitat relationships are strongly scale-dependent [37], [38]. The fact that growth rate models had higher predictive power in the CBC while carrying capacity models had higher predictive power in the BBS might suggest that population growth rate is intrinsically determined at a coarser grain size than carrying capacity. This could be the case if population growth rate were driven in part by immigration from adjoining areas as might be expected with an actively invading species, where coarse-scale landscape configuration and connectivity may be important. Unfortunately, no strong conclusions may be made about the differences in results between BBS and CBC from these analyses alone, but we hope to have highlighted several important avenues of future research.

**Figure 4:**
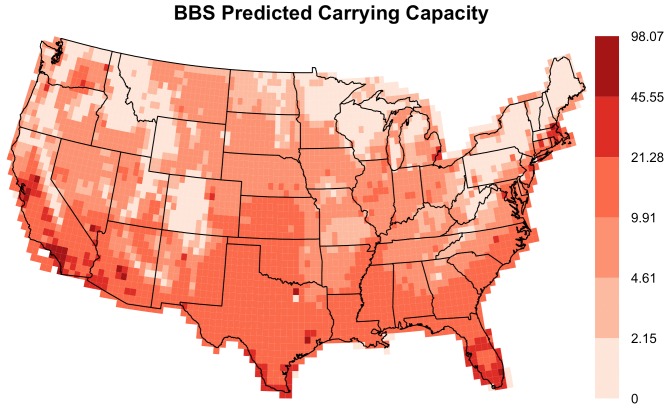
Carrying capacity prediction map. Map of predicted carrying capacity of the Eurasian Collared-Dove across the contiguous United States (Albers Equal Area Conic projection) based on the modeled relationships between Breeding Bird Survey population trajectories and climate and land cover data (see Table 1). Individual cells are 0.5° in each direction, and values represent the expected number of individuals observed on a BBS route within each cell for populations that have reached their carrying capacity.

The models developed here can be used to make predictions about how this invasion will continue to play out across the North American continent (Figure 4). Since low and medium intensity developed cover has been shown to positively affect both carrying capacity and population growth rate, we expect that the ECD will quickly achieve high abundances in suburban areas, especially where agricultural cropland is close at hand (e.g. greater Los Angeles, southern Florida, California Central Valley, Figure 4). In contrast, we expect urban centers, dense forests, mountain ranges and pastureland will limit both abundance and growth rate of individual populations (e.g. Appalachian and Rocky Mountains, and the Dallas metropolitan center, Figure 4). Up to the present, the ECD has been largely absent from the northeastern U.S. [23], despite a seemingly suitable level of development. Greater forest cover throughout this region may be limiting rates of dispersal and colonization. While our model predicts that the ECD will eventually colonize the region more completely, it also predicts that the ECD will not achieve high local abundances there (Figure 4). More targeted research is needed to fully understand this absence, and the continued collection of ECD observations through the BBS, CBC and other programs will eventually put these predictions to the test.

Our examination of population trajectories rather than a single year's abundance pattern provides additional insight into future dynamics over the course of range expansion. Many of the older populations in the southeastern U.S. have seen declines in numbers after initial periods of exponential growth. We expect that many of the sites experiencing rapid population growth at the invasion front may eventually exhibit subsequent declines as well. Another important avenue of future research is to identify the environmental features that determine whether ECDs asymptotically approach local carrying capacity or overshoot it.

Our analysis did not take into account the presence or abundance of other competitors or predators that might impact ECD populations. However, several studies that have examined habitat and dietary overlap of the ECD with native dove species have found only positive associations [19], [39], and more generally some have argued that invasive species only rarely cause the complete competitive exclusion of native species [5], [11], [40]. Thus, invasive species may not interact as strongly with natives as previously thought. Nevertheless, the simultaneous incorporation of both biotic and abiotic variables presents an interesting area of future study with important implications for the modeling and management of range-expanding species.

### Conclusions

Our geographic analysis of population time series across the range of a rapidly expanding invasive species illustrates two points. First, species invasions can be modeled using a predictable population ecology framework that is primarily structured by dispersal, distance and time. Second, landscape features and environment may play a large role in structuring local carrying capacities and population growth rates of an invasive bird species. Future studies of the ECD should attempt to determine the extent to which these population parameters are structured by predation and intraspecific interactions, barriers to dispersal, and food availability. Examining whether these patterns apply to other invasive species will also be important for creating more integrated and informative models of range expansion and invasive spread.

## Supporting Information

Figure S1 **Environmental variable kernel density plots.** Kernel density plot for each environmental variable used in both our carrying capacity and population growth rate analyses for both BBS (orange) and CBC (blue) datasets. The displayed p-values are the result of Mann-Whitney two sample ranked sum tests.
(TIFF)

Figure S2 **Spatial autocorrelation in response variables.** Correlograms of 4 different response variables showing Moran’s I as a function of lag distance (km). Black lines represent raw response values (either carrying capacity or population growth rate), while green lines represent our model averaged residuals and their respective confidence intervals (if they are large enough to be plotted). Zero spatial autocorrelation is represented by the dashed line in each plot.
(TIFF)

Table S1 **Variable relative importance weights and weighted parameter estimates for collectively standardized dataset.** Variable relative importance weights (*w_i+_*) and weighted parameter estimates () based on all multivariate models predicting carrying capacity (*K*) and growth rate (*r*) for both the BBS and CBC datasets. Variables with a relative importance weight greater than 0.7 and their corresponding parameter estimates are highlighted in bold. Coefficient of determination (*R* ^2^) and Akaike weight for the model with the lowest AIC_c_ score are provided for each dependent variable and dataset. Data for the models was drawn from a merged, scaled dataset containing both BBS and CBC data.
(DOCX)
