# Supplementary figures and images for: Range Expansion and Population Dynamics of an Invasive Species: The Eurasian Collared-Dove (Streptopelia decaocto)
Source: PLoS One. 2014 Oct 29;9(10):e111510. doi: 10.1371/journal.pone.0111510 (PMC4213033; doi:10.1371/journal.pone.0111510)

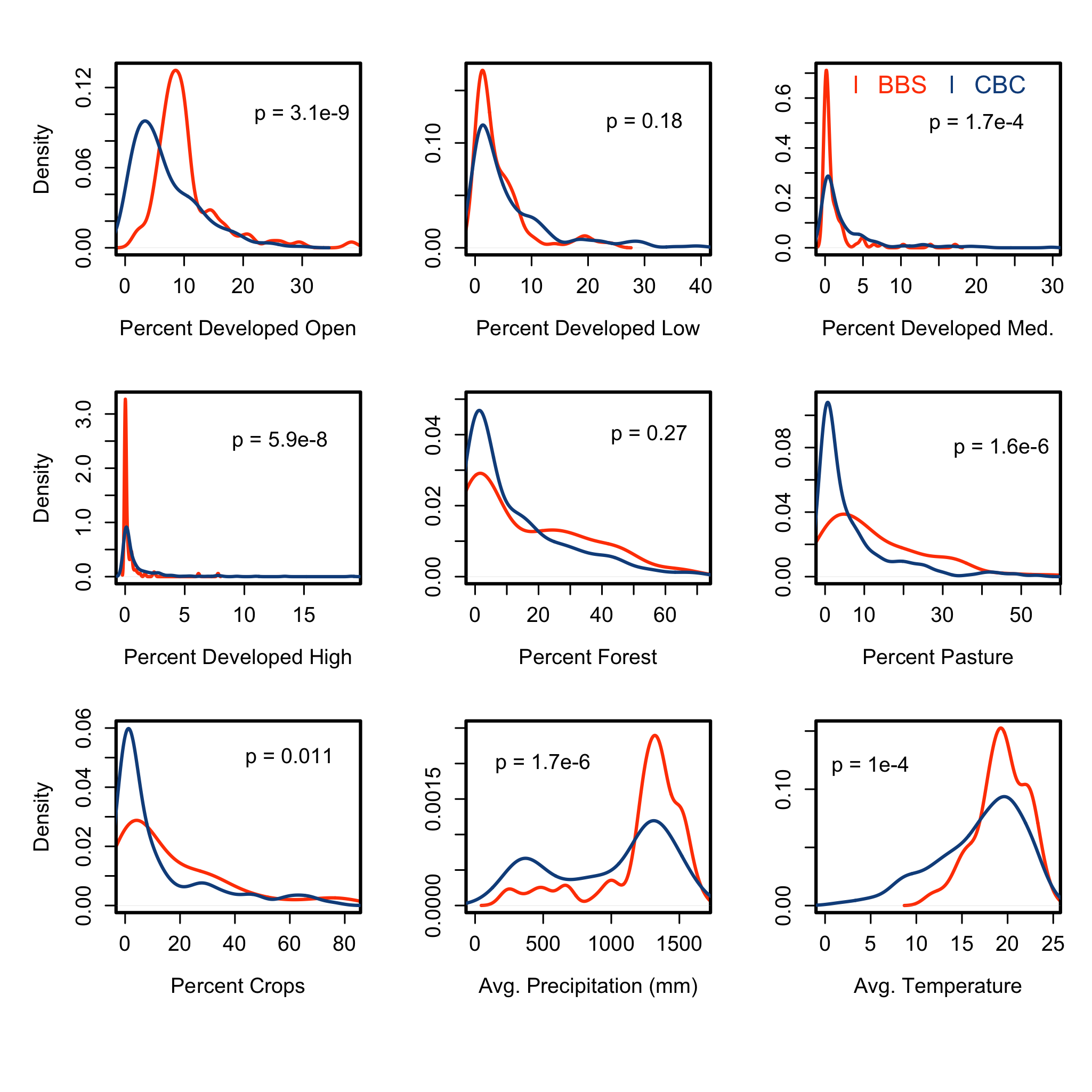

Supplement: Figure S1 — Environmental variable kernel density plots. Kernel density plot for each environmental variable used in both our carrying capacity and population growth rate analyses for both BBS (orange) and CBC (blue) datasets. The displayed p-values are the result of Mann-Whitney two sample ranked sum tests. (TIFF) [file pone.0111510.s001.tiff]

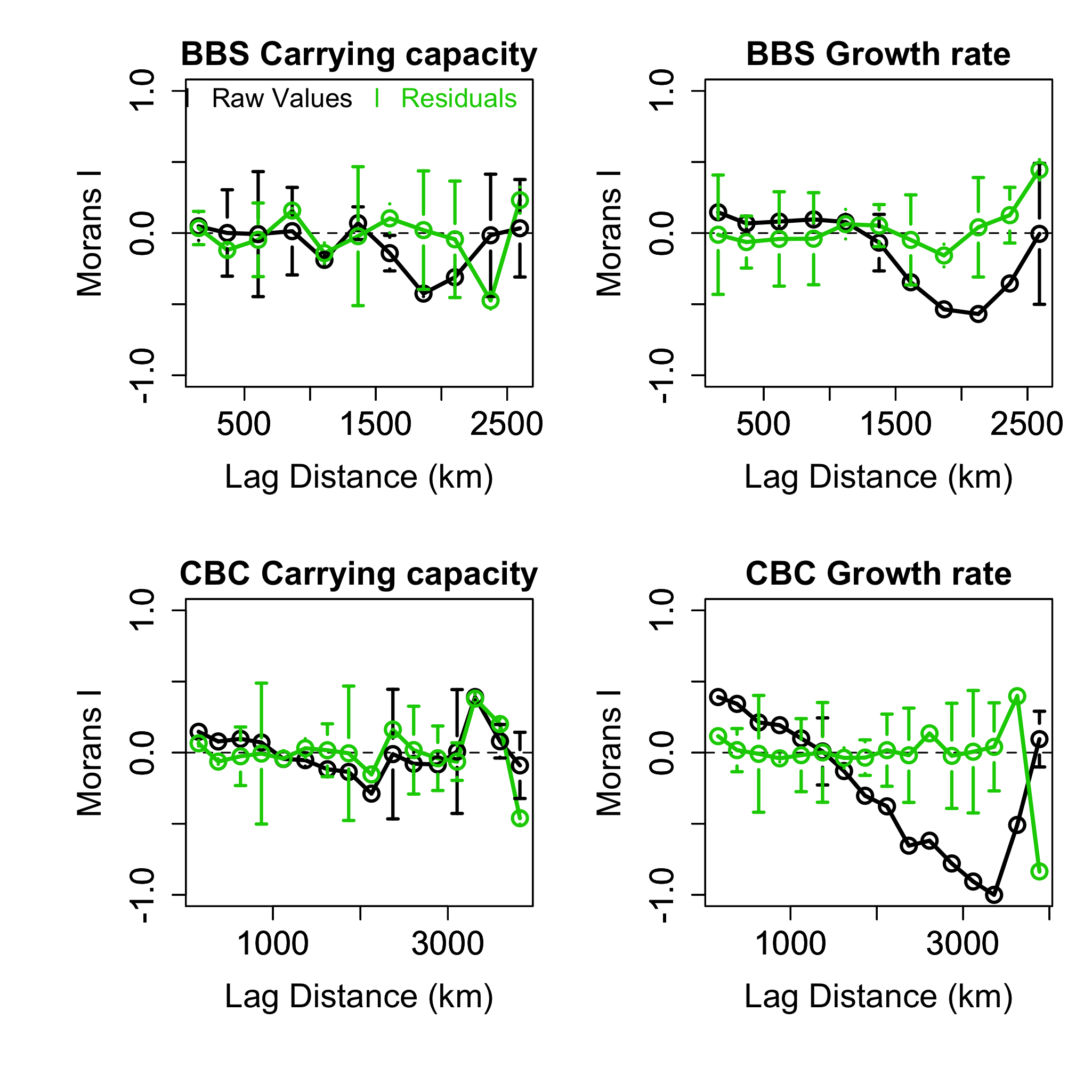

Supplement: Figure S2 — Spatial autocorrelation in response variables. Correlograms of 4 different response variables showing Moran’s I as a function of lag distance (km). Black lines represent raw response values (either carrying capacity or population growth rate), while green lines represent our model averaged residuals and their respective confidence intervals (if they are large enough to be plotted). Zero spatial autocorrelation is represented by the dashed line in each plot. (TIFF) [file pone.0111510.s002.tiff]
