# Supplementary material for: Range Expansion and Population Dynamics of an Invasive Species: The Eurasian Collared-Dove (Streptopelia decaocto)
Source: PLoS One. 2014 Oct 29;9(10):e111510. doi: 10.1371/journal.pone.0111510 (PMC4213033; doi:10.1371/journal.pone.0111510)
Supplement: Table S1 — Variable relative importance weights and weighted parameter estimates for collectively standardized dataset. Variable relative importance weights (wi+) and weighted parameter estimates () based on all multivariate models predicting carrying capacity (K) and growth rate (r) for both the BBS and CBC datasets. Variables with a relative importance weight greater than 0.7 and their corresponding parameter estimates are highlighted in bold. Coefficient of determination (R 2) and Akaike weight for the model with the lowest AICc score are provided for each dependent variable and dataset. Data for the models was drawn from a merged, scaled dataset containing both BBS and CBC data. (DOCX) [file pone.0111510.s003.docx]

**Table S1. Variable relative importance weights and weighted parameter estimates for collectively standardized dataset**

| *r_CBC_* | $\hat{\beta}$ |  | **0.64** | 0.00 |  | -0.01 | **0.14** | 0.03 | 0.04 | -0.07 | 0.00 | 0.01 |  | -0.04 | -0.07 | Variable relative importance weights (*w_i+_*) and weighted parameter estimates ($\hat{\beta}$) based on all multivariate models predicting carrying capacity (*K*) and growth rate (*r*) for both the BBS and CBC datasets and abundance (N) for 2010 CBC sites. Variables with a relative importance weight greater than 0.7 and their corresponding parameter estimates are highlighted in bold. Coefficient of determination (*R*^2^) and Akaike weight for the model with the lowest AIC_c_ score are provided for each dependent variable and dataset. Data for the models was drawn from a merged, scaled dataset containing both BBS and CBC data. | 0.46 |
| --- | --- | --- | --- | --- | --- | --- | --- | --- | --- | --- | --- | --- | --- | --- | --- | --- | --- |
|  | *w_i+_* |  | **1.00** | 0.26 |  | 0.29 | **0.76** | 0.37 | 0.38 | 0.59 | 0.26 | 0.32 |  | 0.35 | 0.57 |  |  |
| *r*_BBS_ | $\hat{\beta}$ |  | **0.45** | -0.02 |  | 0.01 | 0.01 | 0.20 | -0.05 | 0.00 | -**0.14** | -0.02 |  | 0.01 | 0.14 |  | 0.40 |
|  | *w_i+_* |  | **0.98** | 0.31 |  | 0.24 | 0.30 | 0.48 | 0.36 | 0.25 | **0.93** | 0.34 |  | 0.28 | 0.60 |  |  |
| *K*_CBC_ | $\hat{\beta}$ |  | -0.01 | **0.16** |  | -0.05 | 0.06 | 0.01 | 0.03 | -0.01 | -0.02 | **0.11** |  | **-0.21** | **-0.07** |  | 0.35 |
|  | *w_i+_* |  | 0.28 | **0.99** |  | 0.63 | 0.61 | 0.33 | 0.48 | 0.37 | 0.49 | **0.99** |  | **1.00** | **0.76** |  |  |
| *K*_BBS_ | $\hat{\beta}$ |  | -0.06 | -0.01 |  | -0.06 | -0.34 | **2.63** | **-2.11** | -0.12 | -0.08 | 0.00 |  | 0.12 | 0.31 |  | 0.46 |
|  | *w_i+_* |  | 0.29 | 0.23 |  | 0.29 | 0.55 | **0.99** | **0.93** | 0.42 | 0.39 | 0.22 |  | 0.35 | 0.51 |  |  |
|  |  | *Historical/geographical variables* | Distance to invasion origin | Time since colonization | *Land cover variables* | Developed: open cover | Developed: low intensity | Developed: med. Intensity | Developed: high intensity | Forest | Pasture | Crops | *Climate variables* | Avg. Precipitation | Avg. Temperature |  | Model Averaged R^2^ |
